# Integrated Co-functional Network Analysis on the Resistance and Virulence Features in *Acinetobacter baumannii*

**DOI:** 10.3389/fmicb.2020.598380

**Published:** 2020-11-02

**Authors:** Ruiqiang Xie, Ningyi Shao, Jun Zheng

**Affiliations:** ^1^Faculty of Health Sciences, University of Macau, Macau, China; ^2^Institute of Translational Medicine, University of Macau, Macau, China

**Keywords:** *Acinetobacter baumannii*, integrated network, k-shell decomposition, antibiotic resistance, virulence factor

## Abstract

*Acinetobacter baumannii* is one of the most troublesome bacterial pathogens that pose major public health threats due to its rapidly increasing drug resistance property. It is not only derived from clinic setting but also emerges from aquaculture as a fish pathogen, which could pass the resistant genes in the food chain. Understanding the mechanism of antibiotic resistance development and pathogenesis will aid our battle with the infections caused by *A. baumannii*. In this study, we constructed a co-functional network by integrating multiple sources of data from *A. baumannii* and then used the k-shell decomposition to analyze the co-functional network. We found that genes involving in basic cellular physiological function, including genes for antibiotic resistance, tended to have high k-shell values and locate in the internal layer of our network. In contrast, the non-essential genes, such as genes associated with virulence, tended to have lower k-shell values and locate in the external layer. This finding allows us to fish out the potential antibiotic resistance factors and virulence factors. In addition, we constructed an online platform ABviresDB (https://acba.shinyapps.io/ABviresDB/) for visualization of the network and features of each gene in *A. baumannii*. The network analysis in this study will not only aid the study on *A. baumannii* but also could be referenced for the research of antibiotic resistance and pathogenesis in other bacteria.

## Introduction

*Acinetobacter baumannii*, an opportunistic gram-negative bacterial pathogen, has emerged as the most common cause of nosocomial and difficult-to-treat infections (Asif et al., 2018). It has become especially troublesome due to the quick emergence and wide spread of antibiotic resistance. In fact, *A. baumannii* has developed resistance to almost all antibiotics clinically used and the infections by multidrug-resistant strains are very common (Xie et al., 2018; Theuretzbacher et al., 2020). In addition, *A. baumannii* persistence could also contribute to the treatment failure of its infection (Barth Jr., Rodrigues et al., 2013; Zou et al., 2018, 2020; Ji et al., 2019). In fact, death caused by drug-resistant *A. baumannii* infections has increased by approximately 60% in the past 6 years (Allen et al., 2020). The threats by *A. baumannii* have recently been emphasized by WHO, which has listed *A. baumannii* as the priority 1 pathogen for research and development on new antimicrobial treatment (World Health Organization [WHO], 2017). Thus, it is urgent to find new approaches to battle with the infection caused by *A. baumannii*, whereas understanding the factors contributing to its antibiotic resistance will help this endeavor.

In addition to the cause of well-known nosocomial infections, *A. baumannii* has recently been recognized as an emerging fish pathogen, raising the public concern of *A. baumannii* transmission from aquatic culture to human through food chain (Dekic et al., 2018). In fact, *A. baumannii*, including antibiotic resistant *A. baumannii*, has been isolated from a wide range of aquatic environments and diverse fish species (Musa et al., 2008; Xia et al., 2008; Rauta et al., 2011; Brahmi et al., 2016). Behera et al. isolated *A. baumannii* from diseased fish—*Labeo rohita*—and found that intraperitoneal transmission of isolated *A. baumannii* to fish resulted in significant mortality, further confirming that *A. baumannii* is a fish pathogen (Behera et al., 2017). Thus, *A. baumannii* not only threatens human health but also could cause loss in fishery.

To successfully establish a niche in the infected host, bacteria have to be equipped with virulence factors (Cornejo et al., 2017). In addition to the wide existence of drug resistance, the virulence factors of *A. baumannii* contributed greatly to the successful infection in host (Harding et al., 2018; Morris et al., 2019). Biofilm formation, motility, and the secretion systems are common virulence factors for bacterial pathogens, including *A. baumannii* (Harding et al., 2018). Despite extensive research work that has been conducted, the pathogenesis of *A. baumannii* is still yet to be fully understood. Identification and shortlist of potential virulence factors would greatly contribute to the study and understanding on *A. baumannii* infections.

Biological network represents a typically biological system reflecting the direct and indirect connection of different molecular factors. These molecular factors, including genes or proteins, play important roles in the biological process, cellular function, and signaling cascades of the organisms (Vidal et al., 2011). Network topological structure could provide important information from its nodes or edges with the parameters of the centrality, betweenness, and degree. The hub nodes or influential nodes generally play pivotal roles in the complex biological network (Cho et al., 2012) and tend to correspond to proteins that are essential (Zotenko et al., 2008). Recently, the k-shell decomposition method was illustrated to be effective to identify influential nodes in the network (Pei et al., 2014). In general, analyzing the network structure and understanding the topological properties could discover novel nodes in the complex biologic system and provide new insights for elucidation of the molecular mechanism (Yan et al., 2018). For example, through a genome-scale co-functional network analysis, Lee et al. successfully identified several novel genes associated with antibiotic resistance or virulence in *Klebsiella pneumoniae* (Lee et al., 2019).

In this study, we have developed a new strategy to understand the connection of different molecular factors in *A. baumannii*. We integrated the co-expression, operon organization, and the corresponding protein structural data of genes in *A. baumannii* and constructed an integrated network. Our analysis showed that genes involved in basic cellular physiological function, including genes for antibiotic resistance, tended to have high k-shell values and locate in the internal layer of our network. In contrast, the non-essential genes, such as genes associated with virulence, tended to have lower k-shell values and locate in the external of our network. Based on this finding and the protein structure similarity to known resistance and virulence factors, we have identified a list of potential factors involved in antibiotic resistance and bacterial virulence. Furthermore, the *A. baumannii* virulence and resistance database (ABviresDB) was constructed to visualize the network and the features of each gene in *A. baumannii*. The ABviresDB supports several functions for *A. baumannii* research, including searching, browsing, data downloading, feedback, help, and analysis. We expect that our study would contribute to the future research on the antibiotic resistance and virulence of *A. baumannii*.

## Materials and Methods

### Data Source

The genomic data from *A. baumannii* ATCC 17978 were used for our analysis. The gene expression data were sourced from GEO database (Supplementary Table S1). The gene and its promoter as well as the operon information were integrated from the ProOpDB (Taboada et al., 2012) and DOOR2 (Mao et al., 2014) database. The structure family data were extracted from the Superfamily 1.75 (Gough et al., 2001). The pathway information of genes in *A. baumannii* was accessed from KEGG (Kyoto Encyclopedia of Genes and Genomes) database.

### Network Analysis

The centrality in the co-functional network was analyzed using R package igraph (Csardi and Nepusz, 2006). Briefly, the basic features of the co-functional network, including the degree and the number of edges connected to a node, were calculated. Betweenness refers to the number of shortest paths that pass through a node. Closeness means the quantitative index of the centrality of one node. The parameters including degree, betweenness, and closeness were also calculated with the R package igraph.

The k-shell decomposition is a well-established method for analyzing the structure of complex networks (Wei et al., 2015). It assigns an integer index to each node that represents the location of the node in the network based on its connectivity patterns (Carmi et al., 2007). The implementation of the k-shell algorithm was accessed from a previous study and performed in our network analysis with the default cut-off value (Ahmed et al., 2018). The hub node is defined as the node with high value of k shell and high degree. The average degree was calculated by summing the degree of each node and dividing the number of nodes for each shell. The network visualization was applied by R package of networkD3 (Allaire et al., 2017). The network property was analyzed by Cytoscape v3.7.2 (Shannon et al., 2003).

### Statistical Analysis

Linear regression was applied to calculate the relationship between the degree and the k-shell values of nodes by using R program. The functional annotation was used to determine the enrichment of the interested groups of clustered genes. The functional enrichment was carried out by hypergeometric test with DAVID 6.7 (Huang da et al., 2009). The gene expression data of different samples were normalized using Z-score transformation. The co-expression network was constructed based on Pearson’s correlation coefficient for calculating the correlation of two genes. The cut-off of the Pearson correlation coefficient was set as its absolute value of 0.8 for high correlation and *p* < 0.05. The Clusters of Orthologous Groups (COGs) of the protein were predicted by EggNOG database (Huerta-Cepas et al., 2017).

## Results

### The Integrated Regulatory Network in *A. baumannii* Infections

**FIGURE 1:**
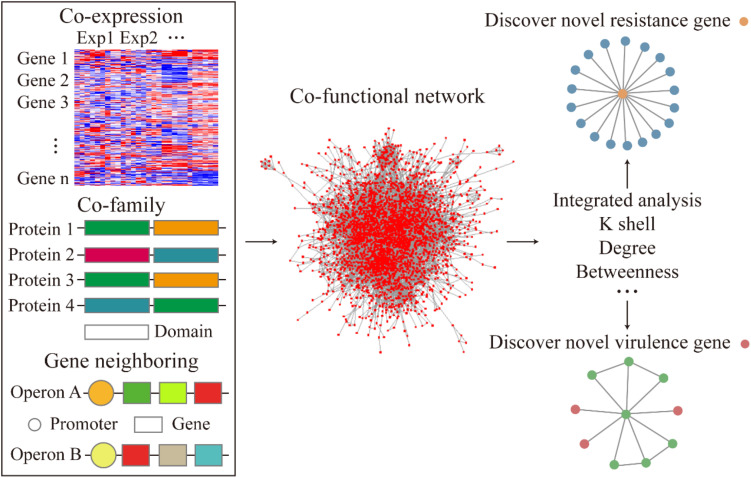
Flow diagram of the co-functional network construction and the analysis of resistance and virulence factors.

**FIGURE 2:**
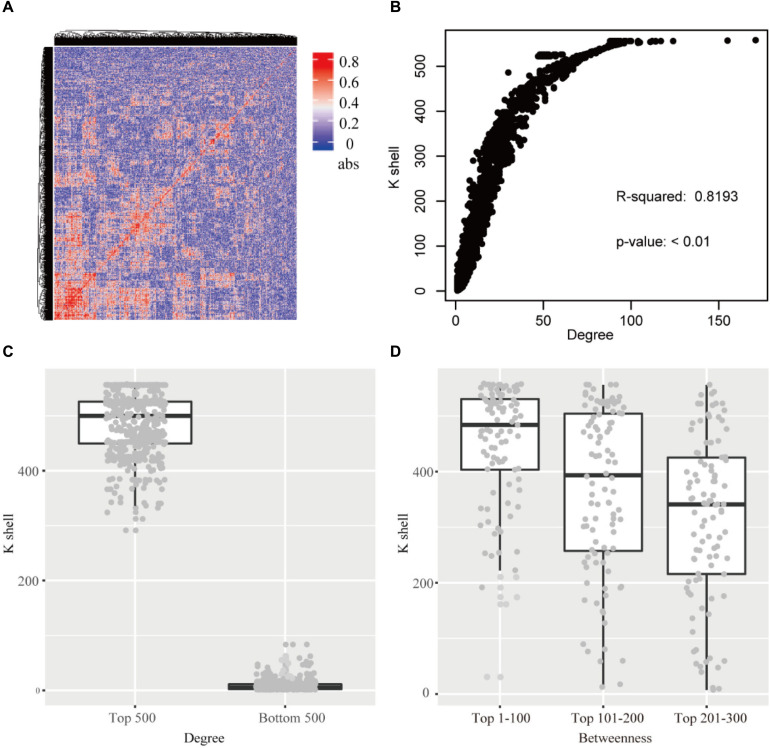
**(A)** The heatmap of the correlation for each gene pair. abs: the absolute value of the correlation. **(B)** The relationship of k-shell value and degree for each gene. **(C)** The genes of top 500 and bottom 500 degree and the corresponding k-shell layers. **(D)** The genes of top 300 betweenness and the corresponding k-shell layers.

To examine the relationship between gene-to-gene interaction at a system level, we curated multiple sources of datasets corresponding to the gene expression, operon organization, and the structural similarities of the corresponding protein to construct a co-functional network (Figure 1). First, to demonstrate the high co-expression relationship between the gene pairs, an absolute value of correlation coefficient, which was greater than 0.8, was set to build the gene co-expression network. The co-expression network was fitted with power-law form (*R*^2^ = 0.892). The heatmap of the correlation coefficient between genes described the whole expression profile of genes in *A. baumannii* (Figure 2A). The gene pairs with highly correlated expression presumably have similar physiological functions or biological features. Next, the operon network was constructed based on the genes from the same operon organization, which demonstrated the co-transcription regulation relationship between genes (Figure 1). Lastly, to find the relationship of the corresponding proteins with a similar structure or the same domain, the protein family encoded by each gene was sourced and integrated to construct a co-family network for gene pairs from the same protein family. Subsequently, we mapped these subnetworks to construct a co-functional network which was fitted with power-law form (*R*^2^ = 0.773, Supplementary Figure S1).

The k-shell decomposition was used to analyze the co-functional network and each gene was assigned to different layers based on the k-shell values. The genes in the internal layers have high k-shell values. In contrast, the genes in the peripheral layers have relatively low k-shell values. We analyzed the top 500 genes based on their k-shell values and found that these genes were mainly clustered in the COGs function of transcription and metabolism that presumably act as the basic physiological function in cell activity (Supplementary Figure S2). In addition, our results found that the k-shell value significantly correlated with its degree (Figure 2B, linear regression *R*^2^ = 0.819). The degree of nodes in internal network has a high value compared with that in the peripheral network (Figure 2C).

We further analyzed the betweenness of the co-functional network and found that the nodes with the top high betweenness were mainly clustered in the internal co-functional network (Figure 2D). The genes with the betweenness on the top 100, which have the high k-shell value of 447 in average, were significantly enriched in the fatty acid biosynthesis pathway (*p* < 0.01, FDR < 0.05).

**FIGURE 3:**
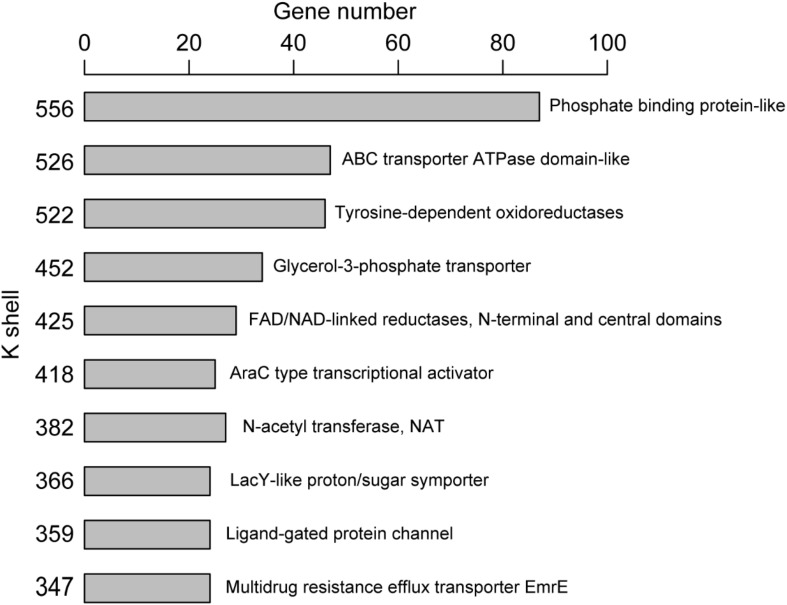
The clustered genes in the same k-shell layer with similar structure.

The majority of genes (or corresponding proteins) having similar structure or function tended to be clustered in the same layer of k-shell decomposition (Figure 3). For example, the genes for proteins with the structure of phosphate binding protein in the k shell of 556 were enriched in ABC transporters pathway (*p* < 0.01, FDR < 0.01). The genes for proteins with the structure of ABC transporter ATPase domain-like, which participated in nucleotide binding and were enriched in ABC transporter pathway (*p* < 0.01, FDR < 0.01), were mainly found in the k shell of 526 (Supplementary Table S2). Nevertheless, we were still able to find that certain genes for proteins with similar structure or function could be clustered in different k-shell layers (Supplementary Figure S3). For example, the genes for proteins with the structure of tetracycline repressor-like domain, which mainly involve in metabolic process and transcription activity, were found in the k shell of 317, 368, and 383 (Supplementary Table S3).

### The Resistance Feature and the Potential Resistance Factors

Antibiotic resistance has become a major concern over the world, especially for *A. baumannii* infection. We analyzed the known resistance factors from the efflux pumps and proteins related drug resistance (Table 1). The genes that have been reported as resistance in literature and that the corresponding proteins associating with the protein family of efflux pump, such as the ATP binding cassette (ABC) transporters, resistance-nodulation-division (RND) transporters, small multidrug resistance (SMR) family, and major facilitator superfamily (MFS), were considered as resistant genes (Abdi et al., 2020b). Such genes were found to have high values of k shell and clustered in the internal layer of the co-functional network (Figure 3). For example, the proteins with the structure of ABC transporter ATPase domain-like were enriched in the k shell of 526. The ABC transporters play an important role in the resistance of *A. baumannii*. The gene of A1S_0536 from ABC transporter pathway, which has a k-shell number of 526, was previously demonstrated to contribute to the resistance to tigecycline, imipenem, and amikacin (Lin et al., 2017). Therefore, other genes, that were enriched in the layer of k shell 526 and that simultaneously the corresponding proteins contain a structure similar to ABC transporter ATPase domain, are likely the potential resistance factors in *A. baumannii* (Supplementary Table S4).

**TABLE 1:** The key resistance genes and selected candidates representing resistance genes in the internal layer of the co-functional network.

Gene name	K-shell	Degree	Betweenness	Product
A1S_0536*	526	49	2,712	Macrolide transport protein
A1S_1242	526	53	9,207	Multidrug ABC transporter transmembrane protein
A1S_3146	366	30	8,106	Multidrug ABC transporter
A1S_1750*	380	30	15,127	AdeB
A1S_2660	314	25	17,889	RND efflux transporter
A1S_0116	246	21	17,817	RND superfamily transporter
A1S_2736	380	30	9,089	RND family drug transporter
A1S_0710	347	26	2,535	SMR family drug transporter
A1S_2298	347	25	0	SMR family efflux pump
A1S_1331*	537	83	72,166	Major facilitator superfamily transporter
A1S_0964	452	37	0	Major facilitator superfamily fosmidomycin/multidrug transport protein
A1S_1516	484	62	47,616	Putative antibiotic resistance
A1S_2198	452	37	0	Putative multidrug resistance protein
A1S_0908	255	18	2,763	RND family multidrug resistance secretion protein
A1S_1772*	366	27	1,546	MFS family transporter
A1S_1773*	255	18	1,544	RND family drug transporter

*Detailed information in Supplementary Tables S4–S8.^∗^Reported resistance factor.*

RND transporters are well known as the major drug efflux pumps in antibiotic resistance (Van Bambeke et al., 2000). The RND transporter AcrB associates with the resistance to a variety of antibiotics (Zgurskaya and Nikaido, 1999; Higgins, 2007). The RND transporter A1S_1750 (AdeB), which contains multidrug efflux transporter AcrB transmembrane domain and was layered in k shell of 380, contributes to multidrug resistance of *A. baumannii* (Abdi et al., 2020a). Thus, it is likely that the genes such as A1S_2736, A1S_2660, and A1S_0116, which have a similar structure of multidrug efflux transporter AcrB transmembrane domain and are simultaneously located in the internal layers of network, are the potential resistance factors (Supplementary Table S5). Similarly, the small multidrug resistance transporter EmrE confers drug resistance to antibiotics (Higgins, 2007). We speculate that the genes with k shell of 347, 358, and 414, which contain the structure of multidrug resistance efflux transporter EmrE, are the potential resistance factors (Supplementary Table S6).

The major facilitator superfamily (MFS) transporters are important factors for antibiotic resistance in *A. baumannii* (Abdi et al., 2020b). The MFS transporter A1S_1331 has a k-shell value of 537, which contains the antibiotic related structure of Glycerol-3-phosphate transporter (Lemieux et al., 2004). It was shown to be responsible for fosfomycin resistance in *A. baumannii* (Sharma et al., 2017). Many genes, which products are predicted to be the MFS transporters, are located in the internal layer of our network. They are likely the potential resistance factors (Supplementary Table S7).

In addition, some genes that were predicted to be the putative resistance factors, multidrug efflux pump and drug transport genes, which were layered in the internal co-functional network, were also considered as potential resistance genes (Supplementary Table S8). For example, the gene A1S_1516 in the k shell of 484 is a putative antibiotic resistance factor and it has a high betweenness parameter (Table 1). It may play a potential important role in the antibiotic resistance in *A. baumannii*.

### The Virulence Feature and the Potential Virulence Factors

*A. baumannii* has become one of the most troublesome pathogens not only because of its resistance property but also its virulence feature. The genes that have been reported to contribute to bacterial virulence in the literature and the genes encoding the related protein family, such as genes for biofilm formation, type II secretion system (T2SS), or type VI secretion system (T6SS), were considered as the virulence factors (Table 2). We found that the virulent factors tended to be in the peripheral layer of the co-functional network (Table 2). For example, the virulence factors A1S_1343, A1S_1193, A1S_2840 (Outer membrane protein A), A1S_1347, and A1S_2989 (Choi et al., 2008; Jacobs et al., 2010; Eijkelkamp et al., 2014; De Silva et al., 2018) are located in the peripheral layer of the network with a k-shell number of 166, 46, 76, 18, and 4, respectively (Table 2). Outer membrane protein A (OmpA) has been studied intensively and well characterized in the virulence of *A. baumannii* (McConnell et al., 2013). We speculate that the genes encoding proteins with a similar structure of OmpA (OmpA-like) and located in the peripheral layer in our co-functional network, such as A1S_1033, A1S_1305, A1S_0884, and A1S_2987, are the potential virulence factors for *A. baumannii* (Supplementary Table S9).

**TABLE 2:** The key virulence genes and selected candidates representing virulence genes in the peripheral layer of the co-functional network.

Gene name	K-shell	Degree	Betweenness	Product
A1S_2840*	76	13	17,992	Outer membrane protein A
A1S_0884	47	8	2,356	Putative outer membrane protein
A1S_2987	46	7	2,247	Putative lipoprotein precursor
A1S_1033	46	7	2,856	Putative antigen
A1S_1193*	46	6	0	OmpA/MotB
A1S_1305	46	7	926	Putative outer membrane lipoprotein
A1S_2989*	4	1	0	Putative phospholipase D protein
A1S_1343*	166	15	3,345	PaaC
A1S_1347*	18	2	0	PaaX
A1S_2213	52	7	8,964	CsuE
A1S_2214	21	4	0	CsuD
A1S_2215	23	6	349	CsuC
A1S_2216	21	4	0	CsuB
A1S_2217	21	5	1,100	CsuA
A1S_2218	48	4	2,992	CsuA/B
A1S_2601	13	4	3,858	Putative outer membrane protein A
A1S_2602	3	1	0	Hypothetical protein

*Detailed information in Supplementary Tables S9–S13.^∗^Reported virulence factor.*

The biofilm formation is strongly associated with the virulence of *A. baumannii*. Therefore, the biofilm formation–related genes (Harding et al., 2018), including A1S_2213, A1S_2214, A1S_2215, A1S_2216, A1S_2217, and A1S_2218, which have k-shell values of 21–48, were considered as the potential virulence factors (Supplementary Table S10).

Furthermore, we analyzed the genes from T2SS or T6SS (Harding et al., 2018). Most of the genes in these two secretion systems were involved in the peripheral layer of the network and considered as the potential virulence factors (Supplementary Tables S11, S12). For example, the genes from T2SS, including A1S_0271, A1S_0270, A1S_0369, A1S_0370, A1S_1563, A1S_1564, and A1S_1565, were layered in k shell of 8, 52, 9, 90, 7, 4, and 74, respectively. The genes from the T6SS, including A1S_0550, A1S_1288, A1S_1289, A1S_1296, and A1S_1310, have a k shell of 9, 7, 22, 32, and 5, respectively.

In addition, the genes of A1S_2601 and A1S_2602, the production of which are from the protein family of virulence factor P.69 pertactin (Emsley et al., 1996) and were layered in k shell 13 and 3, are likely the potential virulence factors (Supplementary Table S13). Outer membrane proteins play important roles in bacteria virulence (Lin et al., 2002). The outer membrane proteins in the peripheral layer of the co-function network might also contribute to virulence (Supplementary Table S13).

### The Online Platform ABviresDB

We constructed an *A. baumannii* virulence and resistance database ABviresDB (see footnote) to illustrate the analyzing results and developing efforts for further research. The database of ABviresDB provides the comprehensive information for the resistance and virulence features through our co-functional network analysis. The candidate of resistance and virulence genes from our analysis could be accessed in ABviresDB.

The features of ABviresDB and its main functions as well as the corresponding statistical information are summarized at its homepage. With the website provided, users can browse all the genes of *A. baumannii* for their detailed information, such as the gene region, gene length, gene product, protein family information, network features, and their candidatures as resistance and virulence factors. Meanwhile, the subnetwork of the interested gene and its connections could be illustrated and shown. Researchers can access the relationship between the interested genes and resistance/virulence factors for further study.

In addition, we provide the browse page for users to choose the interested browsing way and item. The “download” page of the website provides access to data of the genes and our analysis results. Users also can browse the help page for the instructions to use this database. ABviresDB supports the feedback or comments from users to improve our database.

## Discussion

Network analysis is a powerful tool to study complex biological systems, which are normally caused by a combination of genetic and environmental factors (Cho et al., 2012). The development of bacterial resistance involves the complex network of physiological interactions of molecules that adjust the expression profiling. Similarly, bacterial infection is a complex process that coordinates the virulence gene expression at the right environmental circumstance (Crofts et al., 2018). Identification of resistance and virulence factor by traditional molecular technologies is normally labor intensive. Network analysis has been successfully used to analyze the antibiotic resistance and virulence in selected bacteria (Lee et al., 2019). Here, we use the integrated network analysis to construct a co-functional network to investigate the resistance and virulence factors in *A. baumannii*, one of the most threatening bacterial pathogens to humans currently because of its widespread antibiotic resistance. The k-shell decomposition method (Wei et al., 2015) was applied to analyze the co-functional network, and we revealed that genes implicated in basic cellular physiological function, including genes for antibiotic resistance, tended to have high k-shell values and locate in the internal layer of our network. In contrast, the non-essential genes, such as genes associated with virulence, tended to have low k-shell values and locate in the external of our network. Based on the feature of individual genes in the co-functional network, a list of candidates for antibiotic resistance and virulence was proposed.

Antibiotics generally target an essential cellular function of bacteria (Durao et al., 2018). To survive in the presence of such antimicrobials, bacteria have to develop a mechanism to protect themselves from their own antimicrobials or other antimicrobial agents during the evolution (Galan et al., 2013). In fact, bacteria have developed antibiotic resistance at least a few million years ago, long before the antibiotics were discovered and utilized by human beings. The selective pressure of antibiotics drives the evolution of drug resistance in bacteria by *de novo* mutagenesis in the target gene (Durao et al., 2018). In addition, bacteria could also acquire multiple efflux pumps to reduce the intracellular concentration of a drug (Nikaido and Pages, 2012). The concerted action is to prevent the drug from taking effect. Efflux pumps, which were focused by our study, can confer diverse drug resistance as the intrinsic resistance or acquired resistance mechanism, and display broad physiological functions (Sun et al., 2014). For example, an intrinsic resistance determinant KpnGH in *K. pneumoniae*, which is the MFS efflux pump, is involved in crucial physiological functions. Its corresponding mutant demonstrated reduced growth and increased susceptibility to cell envelope stressors (Srinivasan et al., 2014). The involvement of broad physiological functions suggested that the antibiotic resistance genes of efflux pumps may have more connections in the network, which was supported by our observation: the known resistance genes in efflux pumps were hub genes in the internal network (Table 1). This finding is consistent with several previous studies showing that the resistance factors were likely to be the hub nodes or the key connected nodes in the network (Miryala et al., 2019, 2020; Naha et al., 2020). Thus, based on the structural similarity of a protein to efflux pump and its location as a hub gene, we would be able to identify potential resistance factors, which could help us to understand the drug resistance mechanisms in bacteria. The genes closely linked to the resistance factors in the co-functional network might also associate with antibiotic resistance. Those factors that we identified might contribute to antibiotic resistance once the genes are highly induced at certain circumstances or any mutagenesis enhancing their expression level occurs.

Different from antibiotic resistance, the evolution of bacterial virulence was context dependent and highly affected by its host though the host–pathogen interactions (Diard and Hardt, 2017). The process of acquisition or loss of virulence factors involves the interaction of bacteria with or its transmission between its host (Diard and Hardt, 2017). Therefore, the virulence factors may have more links with the external environment. This might explain why virulence factors locate in the peripheral layer of the co-functional network. If the genes in the peripheral layer of the co-functional network associate with known virulence factors, such as T2SS or T6SS, they would have higher chance to be the virulence factors and contribute to the pathogenesis of *A. baumannii*. Therefore, the k-shell value and the localization of gene from our con-functional network would guide experimental research and help find virulence factors for understanding the infection mechanism of *A. baumannii*. Although we are trying to identify novel factors involved in antibiotic resistance or virulence, our analyses were based on the similarity of genes/proteins to known protein architecture that is involved in antibiotic resistance and virulence. Thus, novel factors that have no similarities with such known resistance or virulence factors might not be able to be identified.

In summary, we have integrated the co-expression, operon organization, and structural data of the corresponding protein of gene in *A. baumannii* and built a co-functional network. We discovered that the factors for antibiotic resistance in the bacteria were enriched in the internal layer of the network whereas the virulence factors tended to be in the peripheral layer of the network. This finding could help researchers on *A. baumannii* to fish out the potential antibiotic resistant factors and virulent factors. For such purpose, a database of ABviresDB was developed. The database provides a platform to access the comprehensive information of *A. baumannii* from our analysis, which we expect to help the research work on antibiotic resistance and bacterial pathogenesis of *A. baumannii*. In addition, the approach of network analysis used in our study could also guide the research on antibiotic resistance and pathogenesis in other bacteria.

## Data Availability Statement

The original contributions presented in the study are included in the article/Supplementary Material, further inquiries can be directed to the corresponding author.

## Author Contributions

RX and JZ designed the integrated co-functional network analysis strategy and did the data collection, analysis and interpretation. RX, NS, and JZ drafted the manuscript. All authors provided important intellectual revision of the manuscript.

## Conflict of Interest

The authors declare that the research was conducted in the absence of any commercial or financial relationships that could be construed as a potential conflict of interest.
