# Supplementary material for: Integrated Co-functional Network Analysis on the Resistance and Virulence Features in Acinetobacter baumannii
Source: Front Microbiol. 2020 Nov 2;11:598380. doi: 10.3389/fmicb.2020.598380 (PMC7667040; doi:10.3389/fmicb.2020.598380)
Supplement: Supplementary file 1 [file Data_Sheet_1.pdf]

Integrated co-functional network analysis on the resistance and virulence features in *Acinetobacter baumannii*

Ruiqiang Xie[1], Ningyi Shao[1], Jun Zheng[1, 2,*]

[1] Faculty of Health Sciences, University of Macau, Macau SAR, China;
[2] Institute of Translational Medicine, University of Macau, Macau SAR, China.

Correspondence should be addressed to:
Jun Zheng, junzheng@um.edu.mo

Running title: Network analysis of *A. baumannii* infections

Key words: *Acinetobacter baumannii*; Integrated Network; k-shell decomposition, Antibiotic resistance; Virulence factor

Supplementary Table S1: The raw information of expression sample data from GEO database.

Platforms	GEO accession	Samples
GPL16729	GSE44735	3
	GSE73193	4
	GSE85264	15
GPL19060	GSE67173	3
GPL19074	GSE60348	8
GPL22492	GSE87398	2
	GSE89504	2

Supplementary Table S2: The GO term enrichment of the genes in the layer of k shell 526.

Category	Term	Count	P Value	FDR
MF	GO:0016887~ATPase activity	35	3.58E-42	3.50E-39
MF	GO:0005524~ATP binding	42	1.65E-39	1.61E-36
MF	GO:0032559~adenyl ribonucleotide binding	42	1.98E-39	1.93E-36
MF	GO:0032555~purine ribonucleotide binding	42	6.61E-38	6.44E-35
MF	GO:0032553~ribonucleotide binding	42	6.61E-38	6.44E-35
MF	GO:0030554~adenyl nucleotide binding	42	9.38E-35	9.15E-32
MF	GO:0001883~purine nucleoside binding	42	9.38E-35	9.15E-32
MF	GO:0001882~nucleoside binding	42	1.24E-34	1.21E-31
MF	GO:0017076~purine nucleotide binding	42	1.43E-33	1.39E-30
MF	GO:0000166~nucleotide binding	42	4.01E-30	3.91E-27

MF: molecular function.

Supplementary Table S3: The GO term enrichment of the genes in the layer of k shell 317, 368 and 383 with the structure of tetracycline repressor-like domain.

Category	Term	Count	P Value	FDR
BP	GO:0006350~transcription	27	5.41E-22	3.48E-19
BP	GO:0006355~regulation of transcription, DNA-dependent	27	1.17E-19	7.52E-17
BP	GO:0051252~regulation of RNA metabolic process	27	1.29E-19	8.32E-17
BP	GO:0045449~regulation of transcription	27	1.94E-19	1.25E-16
MF	GO:0003700~transcription factor activity	27	1.27E-23	5.50E-21
MF	GO:0030528~transcription regulator activity	27	2.79E-22	1.21E-19
MF	GO:0003677~DNA binding	27	3.33E-16	1.44E-13

BP: biological process MF: molecular function.

Supplementary Table S4: The ABC transporter family related resistance factors in the internal layer of the co-functional network.

Gene name	Product	K shell	Family description
A1S_2445	high-affinity phosphate transport protein	526	ABC transporter ATPase domain-like
A1S_0003	recombination protein F	526	ABC transporter ATPase domain-like
A1S_0007	putative transport protein	526	ABC transporter ATPase domain-like
A1S_0026	alkanesulfonate transport protein	526	ABC transporter ATPase domain-like
A1S_0144	high affinity Zn transport protein	526	ABC transporter ATPase domain-like
A1S_0321	recombination and DNA repair protein	526	ABC transporter ATPase domain-like
A1S_0344	putative ATP binding site	526	ABC transporter ATPase domain-like
A1S_0433	transport protein Uup	526	ABC transporter ATPase domain-like
A1S_0536*	macrolide transport protein	526	ABC transporter ATPase domain-like
A1S_0647	IcmO protein	526	ABC transporter ATPase domain-like
A1S_0797	putative chromosome segregation ATPases	526	ABC transporter ATPase domain-like; Smc hinge domain
A1S_0899	ATP-dependent dsDNA exonuclease	526	ABC transporter ATPase domain-like; Extended AAA-ATPase domain
A1S_0986	putative transport protein	526	ABC transporter ATPase domain-like
A1S_1059	putative oligopeptide transport protein	526	ABC transporter ATPase domain-like
A1S_1060	putative oligopeptide transport protein	526	ABC transporter ATPase domain-like
A1S_1061	putative oligopeptide transport protein	526	ABC transporter ATPase domain-like
A1S_1240	putative transport protein	526	ABC transporter ATPase domain-like
A1S_1242	multidrug ABC transporter transmembrane protein	526	ABC transporter ATPase domain-like; ABC transporter transmembrane region
A1S_1251	mismatch repair protein	526	ABC transporter ATPase domain-like; DNA repair protein MutS, domain III
A1S_1360	ABC transporter	526	ABC transporter ATPase domain-like
A1S_1361	ABC-type spermidine/putrescine transport system ATPase component	526	ABC-transporter additional domain; ABC transporter ATPase domain-like
A1S_1398	GlnQ protein	526	ABC transporter ATPase domain-like
A1S_1443	taurine ATP-binding transport system component	526	ABC transporter ATPase domain-like
A1S_1483	D-and L-methionine transport protein	526	ABC transporter ATPase domain-like; NIL domain-like
A1S_1493	glutamate/aspartate transport protein	526	ABC transporter ATPase domain-like
A1S_1535	putative transport protein	526	ABC transporter ATPase domain-like; ABC transporter transmembrane region
A1S_1555	lipid transport protein flippase	526	ABC transporter transmembrane region; ABC transporter ATPase domain-like
A1S_1613	putative ABC transporter ATP-binding protein	526	ABC transporter ATPase domain-like
A1S_1671	PltJ	526	ABC transporter ATPase domain-like

A1S_1722	putative ATP-binding component of ABC transporter	526	ABC transporter ATPase domain-like
A1S_1749	ABC transporter-like protein	526	ABC transporter ATPase domain-like
A1S_1785	putative iron transport protein	526	ABC transporter ATPase domain-like
A1S_2073	ABC transporter ATPase	526	ABC transporter ATPase domain-like
A1S_2192	D-and L-methionine transport protein	526	NIL domain-like; ABC transporter ATPase domain-like
A1S_2299	ABC transporter	526	ABC transporter ATPase domain-like
A1S_2311	putative transport protein	526	ABC transporter ATPase domain-like
A1S_2375	putative ABC transporter	526	ABC transporter transmembrane region; ABC transporter ATPase domain-like
A1S_2376	putative ABC transporter	526	ABC transporter ATPase domain-like
A1S_2377	putative ABC transporter	526	ABC transporter ATPase domain-like
A1S_2387	BauE	526	ABC transporter ATPase domain-like
A1S_2536	putative ATPase	526	ABC-transporter additional domain; ABC transporter ATPase domain-like
A1S_2561	hypothetical protein	526	ABC transporter ATPase domain-like; ABC transporter transmembrane region
A1S_2611	transport protein of outer membrane lipoproteins	526	ABC transporter ATPase domain-like
A1S_3084	hypothetical protein	526	ABC transporter ATPase domain-like
A1S_3103	toluene tolerance efflux transporter	526	ABC transporter ATPase domain-like
A1S_3221	putative ABC transporter ATP-binding protein	526	ABC transporter ATPase domain-like
A1S_3295	excinuclease ABC subunit A	526	ABC transporter ATPase domain-like

Note: *: Characterized resistance factor (Lin et al., 2017b)

Supplementary Table S5: The RND family related resistance factors in the internal layer of the co-functional network.

Gene name	Product	K shell	Family description
A1S_2914	preprotein translocase subunit SecD	463	Multidrug efflux transporter AcrB transmembrane domain
A1S_1750*	AdeB	380	Multidrug efflux transporter AcrB transmembrane domain; Multidrug efflux transporter AcrB pore domain; PN1, PN2, PC1 and PC2 subdomains; Multidrug efflux transporter AcrB TolC docking domain; DN and DC subdomains
A1S_2736	RND family drug transporter	380	Multidrug efflux transporter AcrB transmembrane domain; Multidrug efflux transporter AcrB TolC docking domain; DN and DC subdomains; Multidrug efflux transporter AcrB pore domain; PN1, PN2, PC1 and PC2 subdomains
A1S_2660	RND efflux transporter	314	Multidrug efflux transporter AcrB transmembrane domain; Multidrug efflux transporter AcrB TolC docking domain; DN and DC subdomains; Multidrug efflux transporter AcrB pore domain; PN1, PN2, PC1 and PC2 subdomains
A1S_2818	nodulation protein precursor	297	Multidrug efflux transporter AcrB pore domain; PN1, PN2, PC1 and PC2 subdomains; Multidrug efflux transporter AcrB TolC docking domain; DN and DC subdomains; Multidrug efflux transporter AcrB transmembrane domain
A1S_2916	IISP family preprotein translocase membrane subunit	260	Multidrug efflux transporter AcrB transmembrane domain
A1S_0116	RND superfamily transporter	246	oligoketide cyclase/dehydrase-like;Multidrug efflux transporter AcrB transmembrane domain
A1S_0519	hypothetical protein	191	Multidrug efflux transporter AcrB transmembrane domain
A1S_2305	cation/multidrug efflux pump	191	Multidrug efflux transporter AcrB TolC docking domain; DN and DC subdomains; Multidrug efflux transporter AcrB pore domain; PN1, PN2, PC1 and PC2 subdomains; Multidrug efflux transporter AcrB transmembrane domain
A1S_2932	heavy metal efflux pump CzcA	191	Multidrug efflux transporter AcrB transmembrane domain; Multidrug efflux transporter AcrB pore domain; PN1, PN2, PC1 and PC2 subdomains; Multidrug efflux transporter AcrB TolC docking domain; DN and DC subdomains
A1S_3217	RND divalent metal cation efflux transporter	191	Multidrug efflux transporter AcrB transmembrane domain;Multidrug efflux transporter AcrB TolC

			docking domain; DN and DC subdomains; Multidrug efflux transporter AcrB pore domain; PN1, PN2, PC1 and PC2 subdomains
A1S_3445	RND family cation/multidrug efflux pump	191	Multidrug efflux transporter AcrB TolC docking domain; DN and DC subdomains; Multidrug efflux transporter AcrB pore domain; PN1, PN2, PC1 and PC2 subdomains; Multidrug efflux transporter AcrB transmembrane domain
A1S_3446	RND family cation/multidrug efflux pump	191	Multidrug efflux transporter AcrB transmembrane domain; Multidrug efflux transporter AcrB pore domain; PN1, PN2, PC1 and PC2 subdomains; Multidrug efflux transporter AcrB TolC docking domain; DN and DC subdomains

Note: *: Characterized resistance factor (Abdi et al., 2020)

Supplementary Table S6: The SMR family related resistance factors in the internal layer of the co-functional network.

Gene name	Product	K shell	Family description
A1S_2844	quaternary ammonium compound-resistance protein	414	Multidrug resistance efflux transporter EmrE
A1S_2112	putative integral membrane protein	358	Multidrug resistance efflux transporter EmrE
A1S_0163	hypothetical protein	347	Multidrug resistance efflux transporter EmrE
A1S_0164	hypothetical protein	347	Multidrug resistance efflux transporter EmrE
A1S_0254	permease	347	Multidrug resistance efflux transporter EmrE
A1S_0325	hypothetical protein	347	Multidrug resistance efflux transporter EmrE
A1S_0565	hypothetical protein	347	Multidrug resistance efflux transporter EmrE
A1S_0710	SMR family drug transporter	347	Multidrug resistance efflux transporter EmrE
A1S_1097	hypothetical protein	347	Multidrug resistance efflux transporter EmrE
A1S_1253	putative permease	347	Multidrug resistance efflux transporter EmrE
A1S_1323	hypothetical protein	347	Multidrug resistance efflux transporter EmrE
A1S_1505	hypothetical protein	347	Multidrug resistance efflux transporter EmrE
A1S_1712	DMT family permease	347	Multidrug resistance efflux transporter EmrE
A1S_1802	hypothetical protein	347	Multidrug resistance efflux transporter EmrE
A1S_1992	DMT family permease	347	Multidrug resistance efflux transporter EmrE
A1S_2201	hypothetical protein	347	Multidrug resistance efflux transporter EmrE
A1S_2207	membrane protein putative	347	Multidrug resistance efflux transporter EmrE
A1S_2298	SMR family efflux pump	347	Multidrug resistance efflux transporter EmrE
A1S_2502	chloramphenicol-sensitive protein	347	Multidrug resistance efflux transporter EmrE
A1S_2614	hypothetical protein	347	Multidrug resistance efflux transporter EmrE
A1S_3038	hypothetical protein	347	Multidrug resistance efflux transporter EmrE
A1S_3122	hypothetical protein	347	Multidrug resistance efflux transporter EmrE
A1S_3153	hypothetical protein	347	Multidrug resistance efflux transporter EmrE
A1S_3154	hypothetical protein	347	Multidrug resistance efflux transporter EmrE
A1S_3240	hypothetical protein	347	Multidrug resistance efflux transporter EmrE
A1S_3270	putative permease	347	Multidrug resistance efflux transporter EmrE

Supplementary Table S7: The major facilitator superfamily related resistance factors in the internal layer of the co-functional network.

Gene name	Product	K shell	Family description
A1S_1331*	major facilitator superfamily transporter	537	Glycerol-3-phosphate transporter
A1S_1805	major facilitator superfamily transporter	473	Glycerol-3-phosphate transporter
A1S_0161	MFS family transporter	452	Glycerol-3-phosphate transporter
A1S_0193	MFS family transporter	452	Glycerol-3-phosphate transporter
A1S_0775	MFS family transporter	452	Glycerol-3-phosphate transporter
A1S_0909	MFS family transporter	452	Glycerol-3-phosphate transporter
A1S_0915	putative MFS transporter	452	Glycerol-3-phosphate transporter
A1S_0964	major facilitator superfamily fosmidomycin/multidrug transport protein	452	Glycerol-3-phosphate transporter
A1S_1210	major facilitator superfamily transporter	452	Glycerol-3-phosphate transporter
A1S_1257	major facilitator superfamily transporter	452	Glycerol-3-phosphate transporter
A1S_1316	major facilitator superfamily transporter cyanate permease	452	Glycerol-3-phosphate transporter
A1S_1739	major facilitator superfamily transporter	452	Glycerol-3-phosphate transporter
A1S_1867	major facilitator superfamily transporter	452	Glycerol-3-phosphate transporter
A1S_2326	MFS superfamily nitrate transporter transmembrane protein	452	Glycerol-3-phosphate transporter
A1S_2474	major facilitator family transporter	452	Glycerol-3-phosphate transporter
A1S_2754	MFS family transporter	452	Glycerol-3-phosphate transporter
A1S_2860	MFS family transporter	452	Glycerol-3-phosphate transporter
A1S_3440	MFS family transporter	452	Glycerol-3-phosphate transporter

Note: *: Characterized resistance factor (Sharma et al., 2017)

Supplementary Table S8: The Other drug resistance related factors in the internal layer of the co-functional network.

Gene name	Product	K shell	Family description
A1S_2198	putative mutlidrug resistance protein	452	Glycerol-3-phosphate transporter
A1S_1516	putative antibiotic resistance	484	N-acetyl transferase, NAT
A1S_1772*	MFS family transporter	366	LacY-like proton/sugar symporter
A1S_1773*	RND family drug transporter	255	HlyD-like secretion proteins
A1S_0395	Na+-driven multidrug efflux pump	314	
A1S_1799	multidrug efflux MFS transporter putative	366	LacY-like proton/sugar symporter
A1S_2584	major facilitator superfamily family drug transporter	366	LacY-like proton/sugar symporter
A1S_3146	multidrug ABC transporter	366	LacY-like proton/sugar symporter
A1S_0908	RND family multidrug resistance secretion protein	255	HlyD-like secretion proteins

Note: *: Characterized resistance factor (Lin et al., 2017a)

Supplementary Table S9: The OmpA-like related virulence factors in the peripheral layer of the co-functional network.

Gene name	Product	K shell	Family description
A1S_2840*	outer membrane protein A	76	OmpA-like; Outer membrane protein
A1S_0884	putative outer membrane protein	47	OmpA-like
A1S_2987	putative lipoprotein precursor	46	OmpA-like
A1S_1033	putative antigen	46	OmpA-like
A1S_1193*	OmpA/MotB	46	OmpA-like
A1S_1305	putative outer membrane lipoprotein	46	OmpA-like

Note: *: Characterized virulence factor (Choi et al., 2008; Eijkelkamp et al., 2014; De Silva et al., 2018)

Supplementary Table S10: The biofilm formation related virulence factors in the peripheral layer of the co-functional network.

Gene name	Product	K shell	Family description
A1S_2213	CsuE	52	Pilus subunits
A1S_2218	CsuA/B	48	
A1S_2215	CsuC	23	Pilus chaperone
A1S_2217	CsuA	21	
A1S_2216	CsuB	21	
A1S_2214	CsuD	21	

Supplementary Table S11: The Type II secretion systems related virulence factors in the peripheral layer of the co-functional network.

Gene name	Product	K shell	Family description
A1S_0270	putative general secretion pathway protein	52	HtrA-like serine proteases
A1S_0271	putative general secretion pathway protein	8	
A1S_0369	general secretion pathway protein F	9	
A1S_0370	general secretion pathway protein G	90	Pseudopilin
A1S_1563	general secretion pathway protein I precursor	7	GSPII I/J protein-like
A1S_1564	general secretion pathway protein J precursor	4	EpsJ-like
A1S_1565	general secretion pathway protein K	74	GspK pilin-like domain; GspK insert domain-like

Supplementary Table S12: The Type VI secretion systems related virulence factors in the peripheral layer of the co-functional network.

Gene name	Product	K shell	Family description
A1S_0550	putative VGR-related protein	9	Baseplate protein-like; gp4 N-terminal domain-like
A1S_1288	putative VGR-related protein	7	Baseplate protein-like
A1S_1289	putative VGR-related protein	22	
A1S_1296	hypothetical protein	32	Hcp1-like
A1S_1310	hypothetical protein	5	

Supplementary Table S13: The Other virulence related factors in the peripheral layer of the co-functional network.

Gene name	Product	K shell	Family description
A1S_1343*	PaaC	166	HCDH C-domain-like; 6-phosphogluconate dehydrogenase-like, N-terminal domain
A1S_0046	MviN family virulence factor	47	
A1S_2989*	putative phospholipase D protein	4	Phospholipase D; Nuclease
A1S_1347*	PaaX	18	
A1S_2601	putative outer membrane protein A	13	Virulence factor P.69 pertactin
A1S_2602	hypothetical protein	3	Virulence factor P.69 pertactin
A1S_1816	putative long-chain fatty acid transport protein	106	Outer membrane protein transport protein
A1S_2773	putative long-chain fatty acid transport protein	46	Outer membrane protein transport protein
A1S_2325	putative outer membrane protein	32	Outer membrane protein
A1S_0292	putative outer membrane protein W	31	Outer membrane protein
A1S_0708	copper resistance protein B precursor	31	Outer membrane protein
A1S_2075	putative outer membrane protein	31	Outer membrane protein
A1S_2538	outer membrane protein CarO precursor	31	Outer membrane protein
A1S_0693	FilD	23	Outer membrane protein transport protein
A1S_0730	putative long-chain fatty acid transport protein	23	Outer membrane protein transport protein
A1S_0847	putative signal peptide	23	Outer membrane protein transport protein

Note: *: Characterized virulence factor (Jacobs et al., 2010; De Silva et al., 2018)

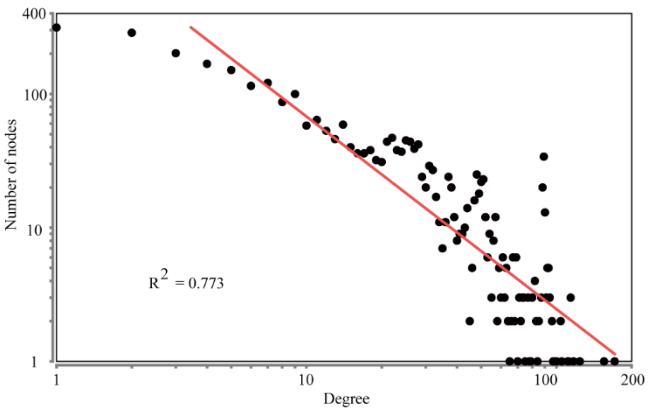

Supplementary Figure S1. The fitted power-law form of the co-functional network.

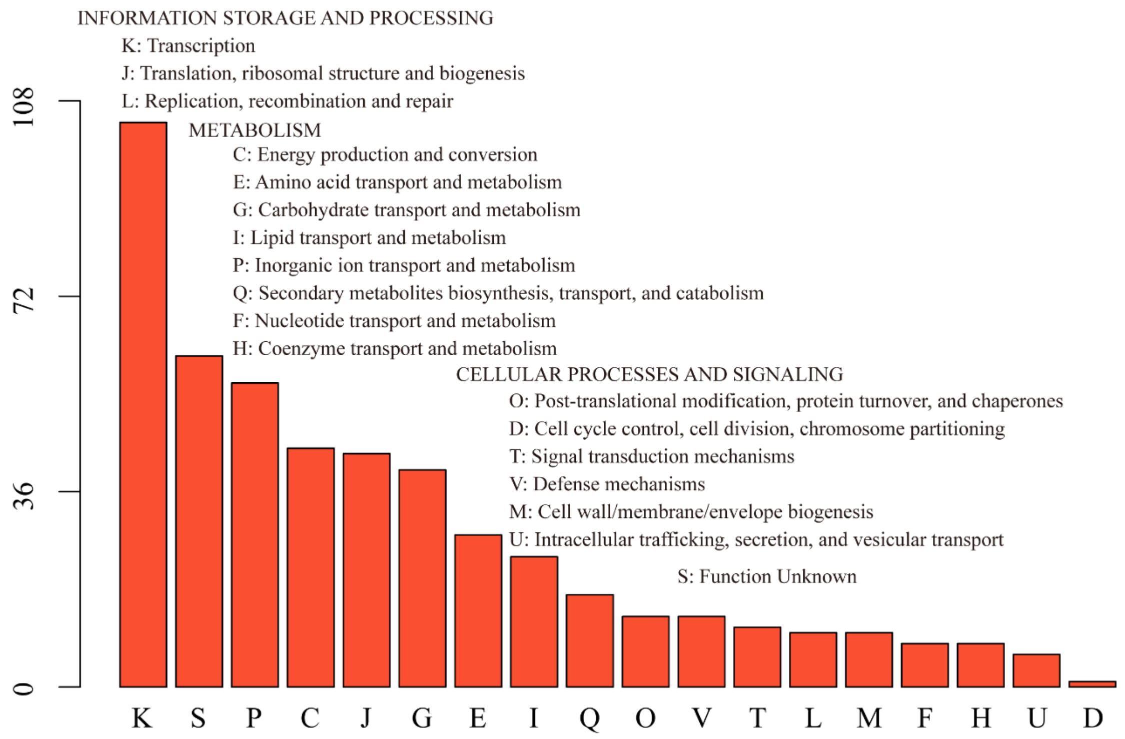

Supplementary Figure S2 The COGs function for the top 500 genes in the internal layer of the co-functional network

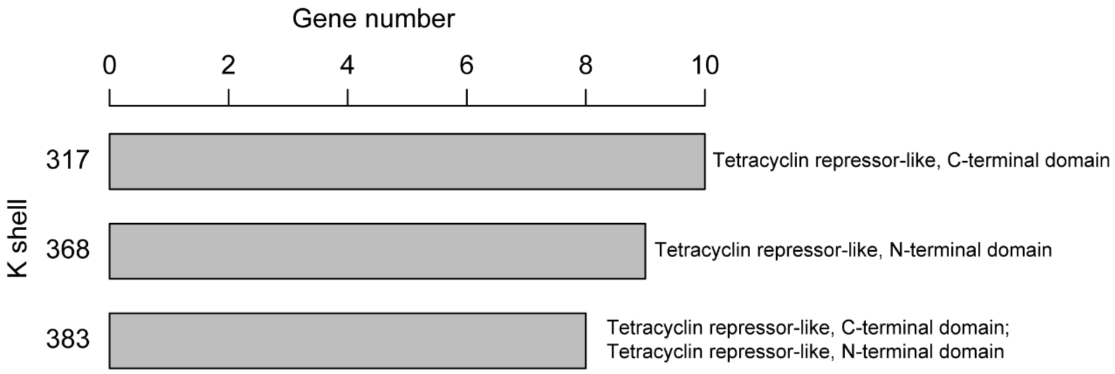

Supplementary Figure S3. The clustered genes in the different k shell layers with similar structure

Supplementary References

Abdi, S.N., Ghotaslou, R., Asgharzadeh, M., Mehramouz, B., Hasani, A., Baghi, H.B., et al. (2020). AdeB efflux pump gene knockdown by mRNA mediated peptide nucleic acid in multidrug resistance Acinetobacter baumannii. *Microb Pathog* 139**,** 103825. doi: 10.1016/j.micpath.2019.103825.

Choi, C.H., Hyun, S.H., Lee, J.Y., Lee, J.S., Lee, Y.S., Kim, S.A., et al. (2008). Acinetobacter baumannii outer membrane protein A targets the nucleus and induces cytotoxicity. *Cell Microbiol* 10(2)**,** 309-319. doi: 10.1111/j.1462-5822.2007.01041.x.

De Silva, P.M., Chong, P., Fernando, D.M., Westmacott, G., and Kumar, A. (2018). Effect of Incubation Temperature on Antibiotic Resistance and Virulence Factors of Acinetobacter baumannii ATCC 17978. *Antimicrob Agents Chemother* 62(1). doi: 10.1128/AAC.01514-17.

Eijkelkamp, B.A., Stroeher, U.H., Hassan, K.A., Paulsen, I.T., and Brown, M.H. (2014). Comparative analysis of surface-exposed virulence factors of Acinetobacter baumannii. *BMC Genomics* 15**,** 1020. doi: 10.1186/1471-2164-15-1020.

Jacobs, A.C., Hood, I., Boyd, K.L., Olson, P.D., Morrison, J.M., Carson, S., et al. (2010). Inactivation of phospholipase D diminishes Acinetobacter baumannii pathogenesis. *Infect Immun* 78(5)**,** 1952-1962. doi: 10.1128/IAI.00889-09.

Lin, M.F., Lin, Y.Y., and Lan, C.Y. (2017a). Contribution of EmrAB efflux pumps to colistin resistance in Acinetobacter baumannii. *J Microbiol* 55(2)**,** 130-136. doi: 10.1007/s12275-017-6408-5.

Lin, M.F., Lin, Y.Y., Tu, C.C., and Lan, C.Y. (2017b). Distribution of different efflux pump genes in clinical isolates of multidrug-resistant Acinetobacter baumannii and their correlation with antimicrobial resistance. *J Microbiol Immunol Infect* 50(2)**,** 224-231. doi: 10.1016/j.jmii.2015.04.004.

Sharma, A., Sharma, R., Bhattacharyya, T., Bhando, T., and Pathania, R. (2017). Fosfomycin resistance in Acinetobacter baumannii is mediated by efflux through a major facilitator superfamily (MFS) transporter-AbaF. *J Antimicrob Chemother* 72(1)**,** 68-74. doi: 10.1093/jac/dkw382.
